# Isolation of Reconstructed Functional Ribonucleoprotein Complexes of Machupo Virus

**DOI:** 10.1128/JVI.01054-21

**Published:** 2021-10-27

**Authors:** Jesse D. Pyle, Sean P. J. Whelan

**Affiliations:** a Department of Molecular Microbiology, Washington University School of Medicine, St. Louis, Missouri, USA; b PhD Program in Virology, Harvard Medical School, Boston, Massachusetts, USA; University of Kentucky College of Medicine

**Keywords:** arenavirus, Machupo virus, RNA-dependent RNA polymerase, gene expression, negative-strand RNA virus, nucleoprotein, replication, ribonucleoprotein complex

## Abstract

Arenaviruses initiate infection by delivering a transcriptionally competent ribonucleoprotein (RNP) complex into the cytosol of host cells. The arenavirus RNP consists of the large (L) RNA-dependent RNA polymerase (RdRP) bound to a nucleoprotein (NP)-encapsidated genomic RNA (viral RNA [vRNA]) template. During transcription and replication, L must transiently displace RNA-bound NP to allow for template access into the RdRP active site. Concomitant with RNA replication, new subunits of NP must be added to the nascent complementary RNAs (cRNA) as they emerge from the product exit channel of L. Interactions between L and NP thus play a central role in arenavirus gene expression. We developed an approach to purify recombinant functional RNPs from mammalian cells in culture using a synthetic vRNA and affinity-tagged L and NP. Negative-stain electron microscopy of purified RNPs revealed they adopt diverse and flexible structures, like RNPs of other *Bunyavirales* members. Monodispersed L-NP and trimeric ring-like NP complexes were also obtained in excess of flexible RNPs, suggesting that these heterodimeric structures self-assemble in the absence of suitable RNA templates. This work allows for further biochemical analysis of the interaction between arenavirus L and NP proteins and provides a framework for future high-resolution structural analyses of this replication-associated complex.

**IMPORTANCE** Arenaviruses are rodent-borne pathogens that can cause severe disease in humans. All arenaviruses begin the infection cycle with delivery of the virus replication machinery into the cytoplasm of the host cell. This machinery consists of an RNA-dependent RNA polymerase—which copies the viral genome segments and synthesizes all four viral mRNAs—bound to the two nucleoprotein-encapsidated genomic RNAs. How this complex assembles remains a mystery. Our findings provide direct evidence for the formation of diverse intracellular arenavirus replication complexes using purification strategies for the polymerase, nucleoprotein, and genomic RNA of Machupo virus, which causes Bolivian hemorrhagic fever in humans. We demonstrate that the polymerase and nucleoprotein assemble into higher-order structures within cells, providing a model for the molecular events of arenavirus RNA synthesis. These findings provide a framework for probing the architectures and functions of the arenavirus replication machinery and thus advancing antiviral strategies targeting this essential complex.

## INTRODUCTION

Arenaviruses (genus *Mammarenavirus*, family *Arenaviridae*, order *Bunyavirales*) are responsible for sporadic outbreaks of rodent-borne hemorrhagic fevers in humans. Notable examples include Lassa fever virus (LASV), which is responsible for an estimated 5,000 to 10,000 deaths, annually (1), and the Junin, Machupo (MACV), and Guanarito viruses, which cause Argentine, Bolivian, and Venezuelan hemorrhagic fevers, respectively (2). Lymphocytic choriomeningitis virus (LCMV), a model for arenavirus biology and mapping of the cellular immune responses, is globally seroprevalent and responsible for febrile illness, aseptic meningitis, neonatal disease, and multiple organ failure in transplant patients (3–9). Although promising vaccine candidates (10–13) and therapeutic treatments (14–16) have been developed to combat arenavirus diseases, to date there are limited effective intervention strategies for infected individuals.

**FIG 1:**
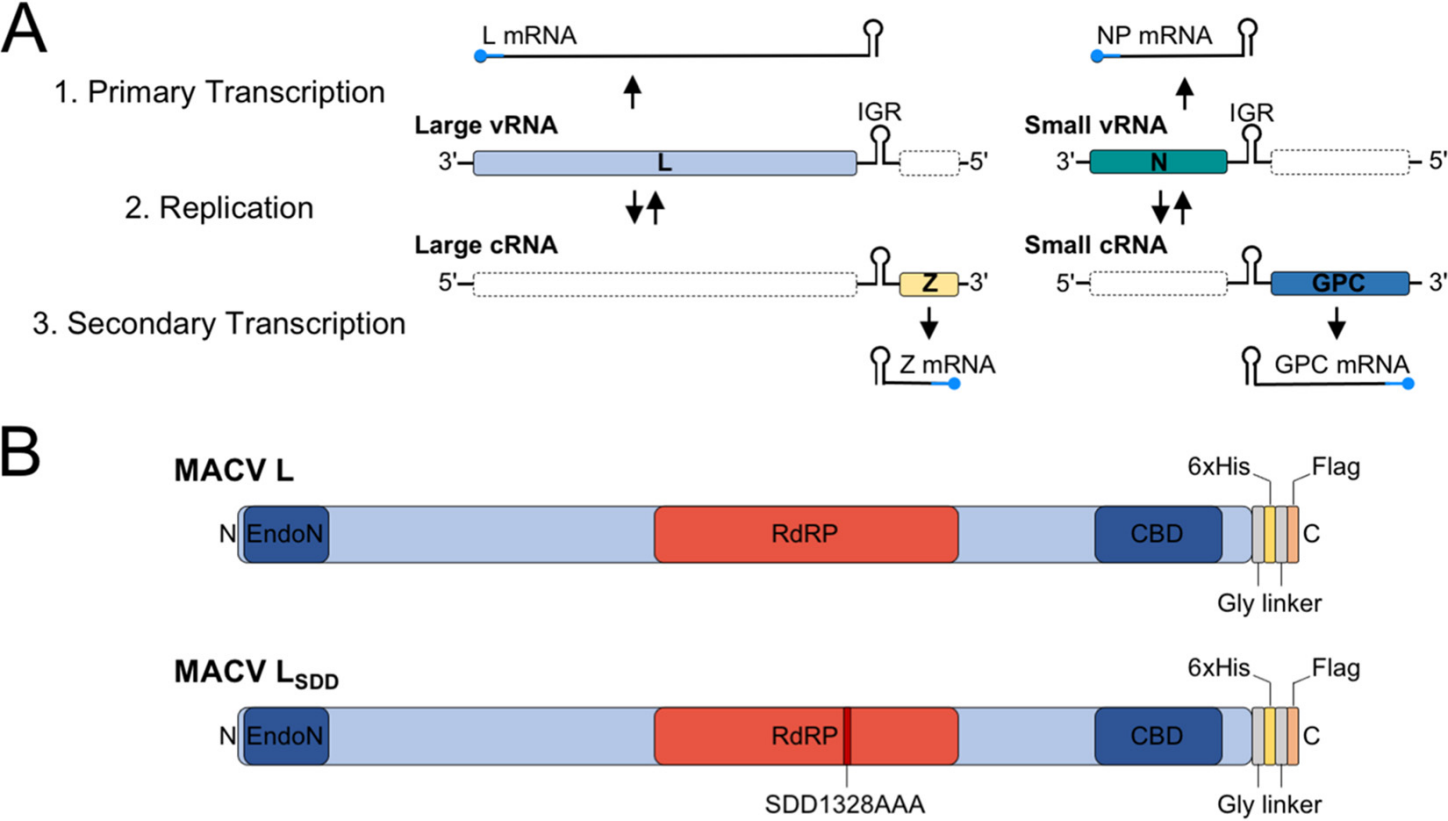
Arenavirus genome organization and gene expression strategies. (A) Illustration of the mammarenavirus genome and sequential stages of transcription and replication during the infection cycle. The sequential order of primary transcription (L and NP mRNA synthesis), genome replication (cRNA synthesis from vRNA template), and secondary transcription (Z and GPC mRNA synthesis) are listed numerically on the left-hand side. Viral ORFs in the expressed coding orientation are shown on corresponding template RNAs as colored boxes, whereas the antisense ORFs are illustrated as empty white boxes with a dashed outline. 5′ cap structures are shown as rounded-end caps on the mRNAs and are colored blue to indicate nonviral sequences acquired via cap snatching. The intergenic regions (IGRs) of each RNA segment are illustrated between the ORFs as a structured hairpin cartoon. (B) Linear map of the Machupo virus (MACV) L protein. The C-terminal cap-binding domain (CBD) and N-terminal cap-dependent endonuclease (EndoN) are shown in dark blue. The central RNA-dependent RNA polymerase (RdRP) domain is shown in red. The introduced affinity tags described in this study are shown as colored regions at the C terminus of L. The position of the mutated residues (SDD1328AAA) for the catalytically inactive mutant of L (LSDD) is shown in dark red.

The arenavirus genome consists of two negative-strand RNA segments encoding four viral proteins: a large (L) RNA-dependent RNA polymerase (RdRP) and matrix protein (Z) within the larger RNA segment, and an RNA-binding nucleoprotein (NP) and glycoprotein precursor (GPC) within the smaller RNA segment (Fig. 1A). The genome-sense RNAs (viral RNAs [vRNAs]) are fully encapsidated by NP subunits and bound at both genome termini by L to form pseudocircularized ribonucleoprotein (RNP) complexes (17–19). Genomic RNPs (viral RNPs [vRNPs]) are enveloped by a lipid bilayer decorated with mature viral glycoproteins, forming the infectious arenavirus virion.

Upon glycoprotein-mediated virion fusion within acidified endosomes, the packaged vRNPs are delivered into the host cytoplasm to initiate viral gene expression and genome replication. Viral mRNA synthesis begins with cap-snatching, whereby the L cap-binding domain recognizes 5′ m^7^GpppN cap structures of host mRNAs for L-mediated endonucleolytic cleavage and priming of transcription initiation (20–29). As L transcribes along the vRNA, NP is transiently displaced to allow template access into the RdRP catalytic site. The growing mRNA is not bound by NP and terminates at highly structured intergenic regions (IGRs) between the two open reading frames (ORFs) of the vRNA segments (Fig. 1A) (28, 30–36).

L and NP mRNAs are the first transcripts synthesized from the large and small RNA segments, respectively (Fig. 1A). L and NP, provided with a suitable RNA template, are the minimal transactivating factors required to initiate arenavirus genome replication (34, 37, 38). After a single round of vRNA copying into antigenomic cRNA, a secondary round of transcription is required for expression of the Z and GPC mRNAs. Z directly mediates catalytic shutdown of L activity (39–44), and GPC is required for infection of new cells. cRNAs are also encapsidated by NP to form cRNPs; however, these replication intermediates are selectively excluded from budding virions (45–50).

Individual NP subunits fold into a two-lobed structure with defined N- and C-terminal functional domains. Each NP monomer binds 6 to 7 nucleotides (nt) of RNA via positively charged residues in a groove formed by the N-terminal domain (51–53). RNA binding is controlled by a gating mechanism, requiring a switch from “closed” to “open” conformations and removal of obstructing alpha helices from the binding pocket to allow for RNA recognition (52, 54). RNA binding induces rearrangement of the NP C terminus, allowing for head-to-tail NP oligomerization along the RNA (55). The C-terminal region of NP is responsible for RNP incorporation into budding virions at the cell membrane via binding of Z (55, 56). Binding of the highly structured IGR sequences by NP is thought to suppress L termination within this region during mRNA synthesis (28, 35, 36), further underscoring the essential role of NP in arenavirus transcription and replication (34). Arenavirus NP is phosphorylated by a virion-associated host kinase (57), and transient phosphorylation of a conserved threonine residue in the RNA binding domain regulates replication-transcription complex (RTC) puncta formation in the host cell cytoplasm (58, 59).

Besides encapsidation of vRNA and cRNA segments, arenavirus NPs have additional effector functions during infection—primarily evasion of the innate immune responses of the cell. NP contains a C-terminal 3′→5′ DEDDh exonuclease (ExoN) fold with specificity for immunostimulatory double-stranded RNAs (dsRNAs) of viral origin (53, 54, 60–63). Degradation of dsRNAs—pathogen-associated molecular patterns (PAMPs) recognized by infected host cells as robust inducers of innate immune signaling pathways—prevents antiviral signaling by cytosolic RNA sensors such as retinoic acid-inducible gene I (RIG-I) or melanoma differentiation-associated protein 5 (MDA5) (64–66). The NP ExoN of Old World mammarenaviruses (i.e., LASV) eliminates immunostimulatory dsRNAs from infected cells more efficiently than that of New World mammarenaviruses (e.g., MACV and Junin virus), revealing a divergence in the immune-evading functions of NP (67, 68), possibly reflecting fundamental differences in the ExoN activity of some viral NPs. In agreement with this, the isolated C-terminal domain of the Junin virus NP does not exhibit ExoN activity *in vitro* (69).

As the sole protective coating for viral genomic and antigenomic RNA templates, the functions of negative-strand RNA virus (NSV) nucleoproteins are tightly linked to the activities of the viral polymerase. An affinity balance is required between polymerase-nucleoprotein and nucleoprotein-RNA binding and the ability of the polymerase to displace nucleoprotein subunits from the RNA during elongation. Arenavirus L can interact with NP indirectly via vRNA scaffolds and by direct binding (70–73). However, the precise mechanisms and biological significance of these interactions remain almost entirely unclear.

The subcellular complexes formed by L and NP during arenavirus infections remain unknown. Currently, our structural understanding of the arenavirus RTC is limited to electron microscopy (EM) observations of virion-associated RNPs. Here, we provide insight into the diverse intracellular structures formed by the polymerase and nucleoprotein of Machupo virus by reconstitution of functional MACV RNPs in hamster cells. MACV L and NP with engineered affinity tags assemble to form replication- and transcription-competent RNPs within cells. These complexes were purified and characterized by negative-stain EM. The MACV complexes adopt diverse structures ranging from monomeric L-NP particles, homotrimeric NP ring-like assemblies, and higher-order flexible filamentous nucleocapsids. These findings provide the first structural insights into the subcellular organization of actively replicating arenavirus L and NP subunits. Moreover, these findings highlight the utility of such reconstituted RNP systems for studying the replication machinery of highly pathogenic mammarenaviruses.

## RESULTS

### Affinity-tagged MACV L and NP assemble into functional RNPs.

**FIG 2:**
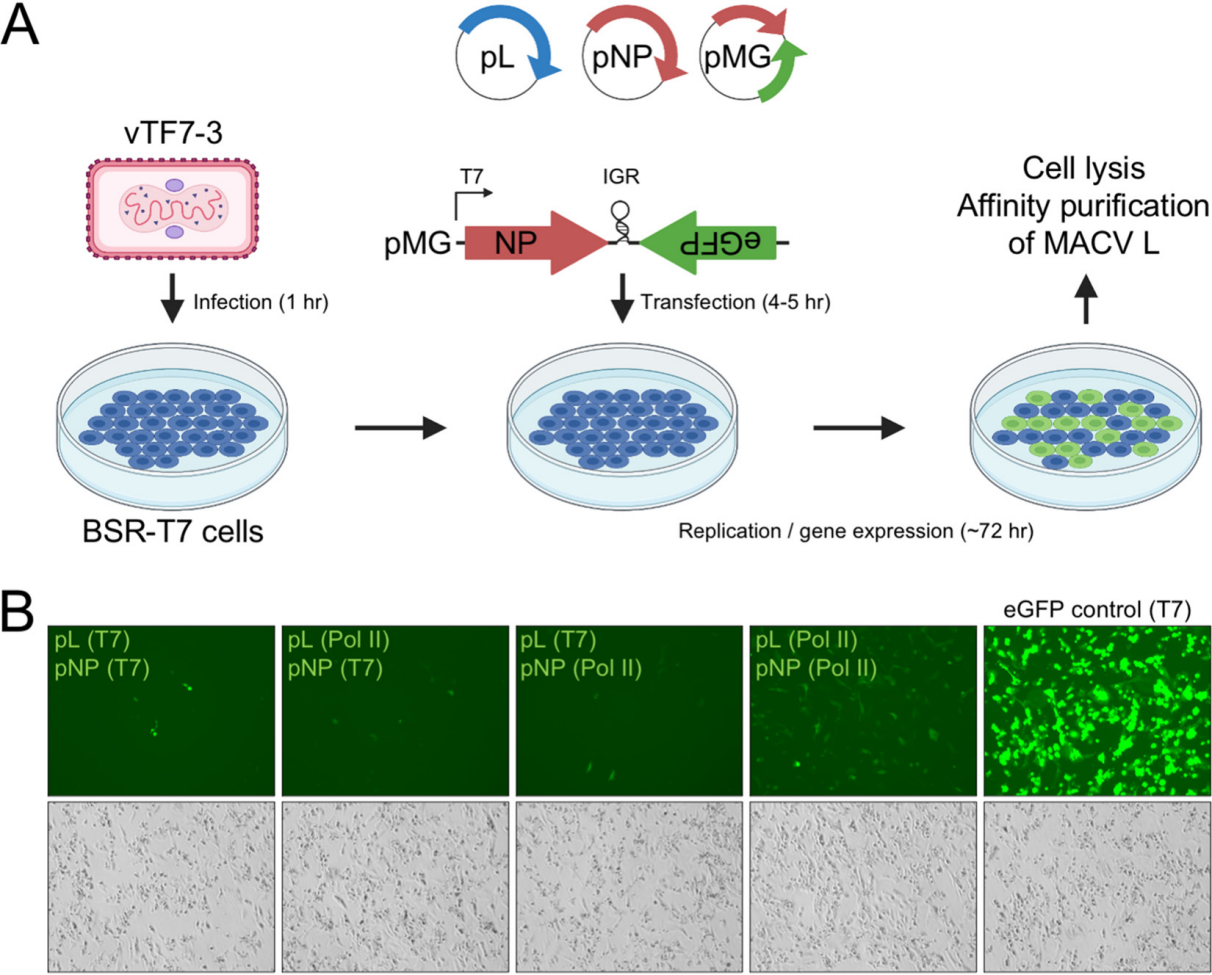
Expression of affinity-tagged L in the MACV replicon system. (A) Schematic of the MACV minireplicon system. (B) Analysis of minireplicon efficiency for L and NP expressed via different promoters (T7 or Pol II). Fluorescence microscopy images of the eGFP-expressing cells are shown in the upper panel. Bright-field images of infected and transfected cells in the same field of view are shown below. Cells transfected with a plasmid encoding T7 RNAP-driven eGFP are shown in the right-hand column.

**FIG 3:**
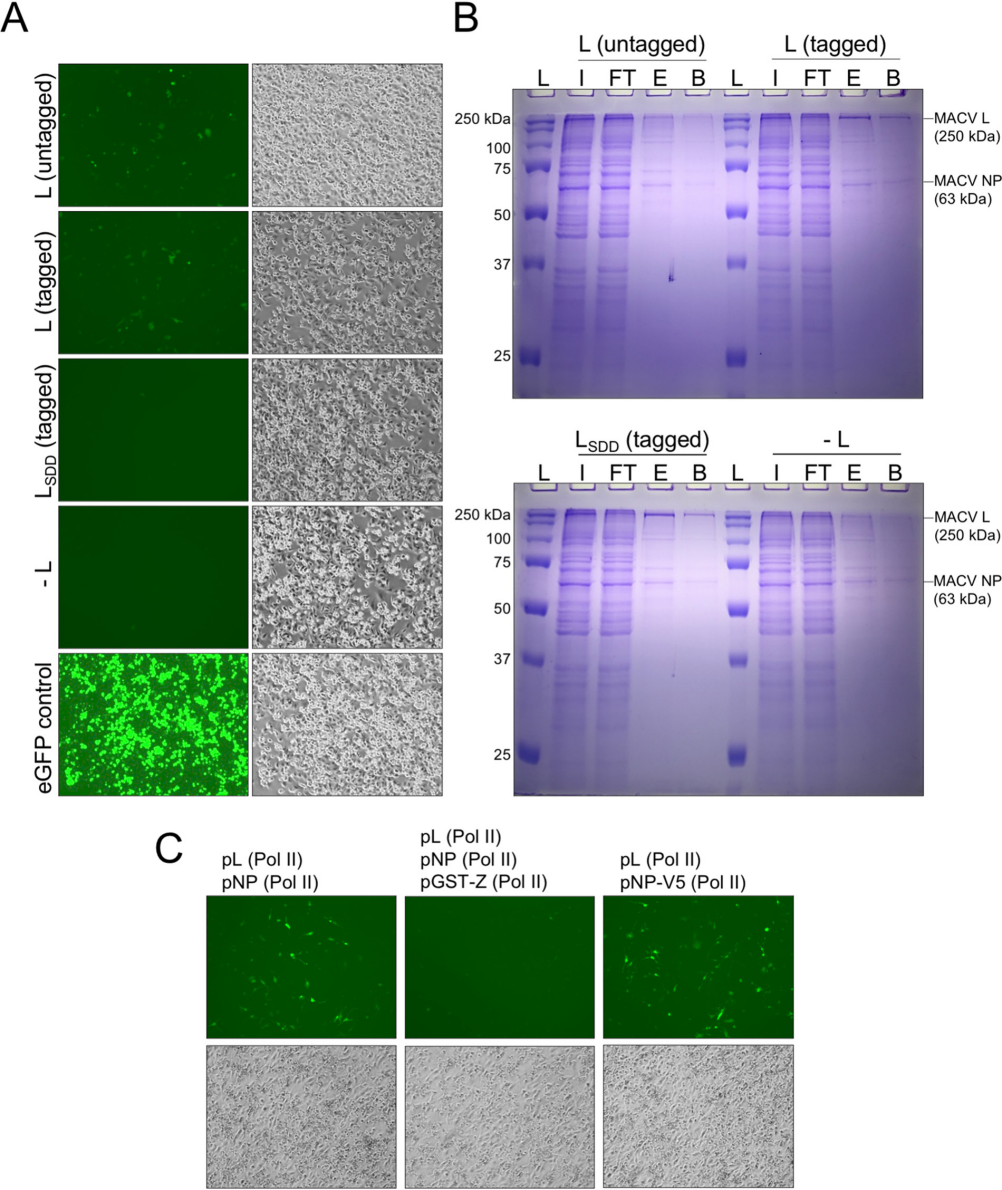
Further characterization of affinity-tagged L and NP in the MACV RNP system. (A) Comparison of minireplicon replication and gene expression mediated by affinity-tagged MACV L in BSR-T7 cells. Bright-field images of infected and transfected cells are shown in the left-hand column. Fluorescence microscopy images of the eGFP-expressing cells in the same field of view are shown in the right-hand column. Cells were transfected with the minireplicon RNA (pMG) and nucleoprotein-expressing (pNP) plasmids, in combination with either wild-type L (untagged), L with C-terminal Flag and 6×His tags (tagged), catalytically inactive L with C-terminal tags (L_SDD_; tagged), or without any L-expressing plasmid (−L). Cells transfected with a plasmid encoding T7 RNAP-driven eGFP expression are shown in the bottom panel. (B) Ni-NTA purification of MACV L from RNP-expressing cells. Cells in panel B were collected and subjected to metal ion affinity purification as described in Materials and Methods. The sample fractions for each condition include the total cell lysate input (I), unbound flowthrough (FT), eluted sample (E) in 0.25 M imidazole, and residual bound proteins from the boiled Ni-NTA beads (B). Lane L contains the protein molecular weight ladder. (C) Further characterization of the modified RNP system with Pol II-expressed L and NP with introduced affinity tags. All cells in panels A and B were infected with T7-expressing vaccinia virus for expression of the minireplicon RNA prior to plasmid transfection.

To isolate actively replicating and transcribing MACV L from relevant host cells, we adapted our previously developed T7 RNA polymerase (T7 RNAP)-driven minireplicon system to incorporate affinity tag epitopes into the L polypeptide (74) (Fig. 1B). This system relies on expression of a modified MACV small RNA segment, with enhanced green fluorescent protein (eGFP) replacing the antisense GPC ORF (Fig. 2A). Consequently, when expressed with MACV L in *trans*, eGFP production is an indicator of L-mediated RNA synthesis activity (74). Insect cell-expressed MACV L retains *in vitro* promoter initiation and elongation activities with added C-terminal affinity tags (39, 74, 75). Thus, we reasoned that similar L modifications could be amenable for the cell-based replicon system and affinity purification of MACV L from mammalian cells. The T7 RNAP expression plasmids for wild-type and catalytically inactive (L_SDD_) MACV L were modified by incorporating Flag (DYKDDDDK) and 6×His (HHHHHH) affinity tags at the C terminus of L for antibody and metal affinity protein purification, respectively (Fig. 1B). These added epitopes did not affect the ability of L to recognize a minireplicon RNA as a suitable template for replication and transcription within cells (Fig. 3A). Preliminary nickel affinity purifications revealed that L could be isolated from RNP-expressing BSR-T7 cells in a 6×His tag-dependent manner, with the most abundant copurifying protein migrating at the approximate expected size of MACV NP (∼63 kDa) (Fig. 3B). These results demonstrate that affinity-tagged MACV L can function as a replicase and transcriptase in a cell-based functional RNP system and that L predominantly coelutes with NP in a single-step metal ion affinity purification strategy.

Visually, the fluorescence intensity and percentage of eGFP-positive cells were very low compared to those of T7 RNAP-driven transfection controls (Fig. 3A), and this was reflected in the low abundances of purified protein (Fig. 3B). This is in line with our original observations for the MACV RNP system, where low numbers of eGFP-positive cells were observed relative to the total number of transfected cells (74). To address this issue, we analyzed different promoter combinations for MACV L and NP expressed in *trans* with the minireplicon RNA, as has been done for other arenavirus replicon systems (76, 77). Synthesis of transcripts by vaccinia virus-expressed T7 RNAP results in high levels of cytoplasmic protein production in cells (78, 79), which could be problematic considering the enzymatic activities and potential cytopathic effects of L and NP overexpression in cells (EndoN, ExoN, RdRP, RNA binding, and 5′ cap binding). We found that expression of MACV L and NP from plasmids with host RNA polymerase II (Pol II) promoters resulted in greater numbers of eGFP-positive cells (Fig. 2B). Under each condition, the cells were infected with vaccinia virus to allow T7-driven expression of the minireplicon RNA. Furthermore, we demonstrated that eGFP expression from the minireplicon is inhibited by expression of a glutathione *S*-transferase (GST)-tagged MACV Z protein (Fig. 3C), as described using the T7 RNAP-based original cell-based replicon system (39). Moreover, expression of NP with a C-terminal V5 epitope tag (GKPIPNPLLGLDST) resulted in successful RNP replication and transcription, opening potential avenues for further affinity purification or in-cell imaging of RTCs (Fig. 3C). Together, these findings demonstrate that affinity-tagged L and NP can assemble functional RNPs and operate more efficiently when expressed from Pol II-driven promoters instead of promoters for vaccinia virus-expressed T7 RNAP.

### NP immunoprecipitates with actively replicating L.

**FIG 4:**
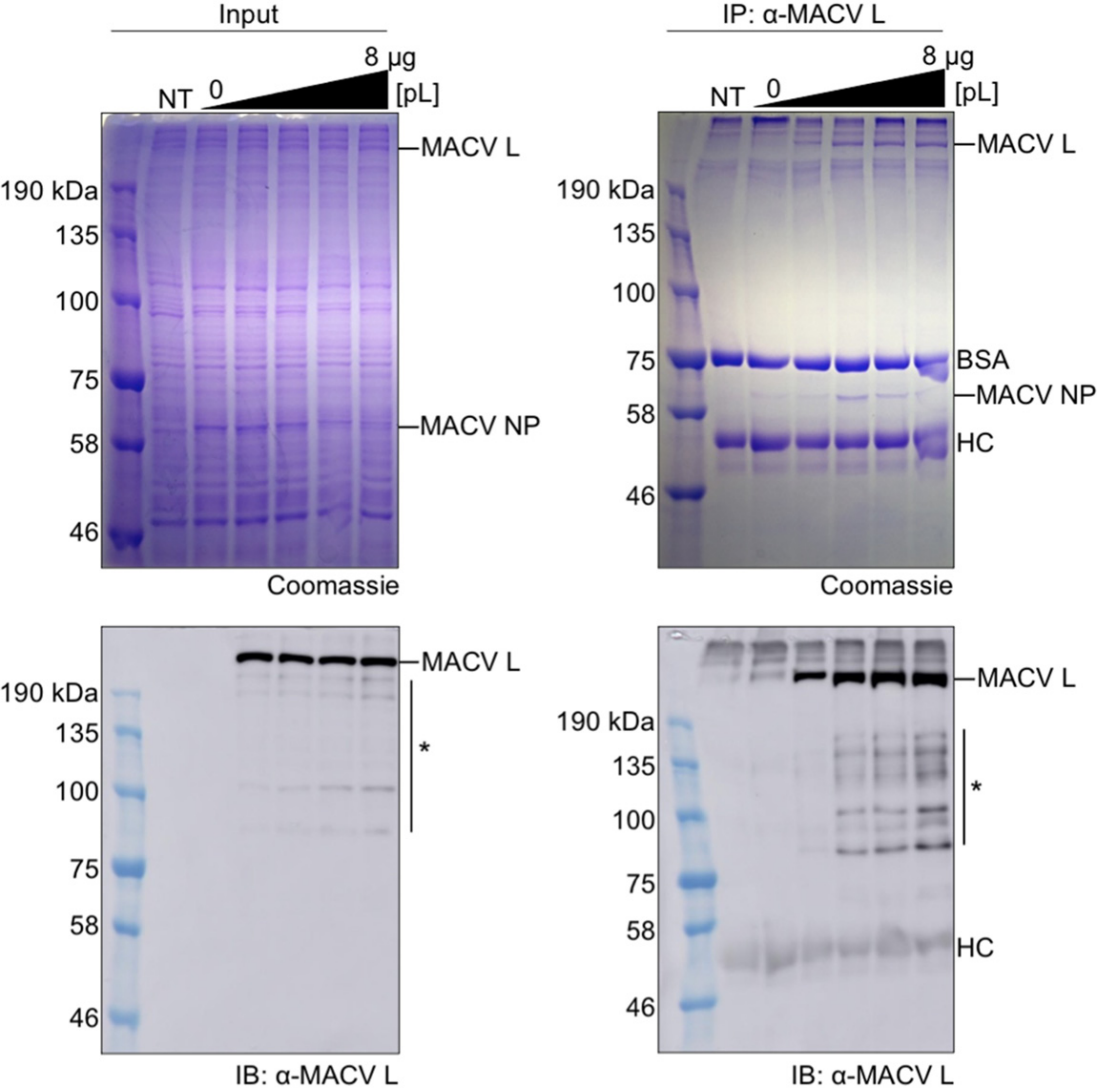
L and NP coimmunoprecipitate from RNP-expressing cells. Polyacrylamide-SDS gels with total proteins from the cell lysate input (left) and immunoprecipitated complexes (right) were run in parallel for Coomassie staining (top) and immunoblotting analyses (bottom). Amounts of pL plasmid transfected into cells included 0, 2, 4, 6, and 8 μg corresponding to the last five lanes of each gel. Truncated or nonspecific proteins detected by the anti-MACV L polyclonal antisera are illustrated with an asterisk (*). BSA, bovine serum albumin; HC, heavy chain of the anti-MACV L antibodies; NT, nontransfected negative-control cells.

We next attempted to optimize MACV L expression in RNP-expressing cells for subsequent purification strategies. Vaccinia virus-infected BSR-T7 cells were transfected with plasmids encoding T7 RNAP-expressed MACV minireplicon RNA (pMG) and transacting Pol II-expressed L and NP (pL and pNP). The mass of pL transfected into cells was tested over a range of 0 to 8 μg. MACV L was immunoprecipitated from lysates of transfected cells using a rabbit polyclonal antibody raised against recombinant L purified from insect cells. The relative abundance of immunoprecipitated and cell lysate-associated L was determined by SDS-PAGE, Coomassie blue staining, and immunoblotting. Immunoprecipitated MACV L reached saturation at approximately 6 μg of transfected pL plasmid (Fig. 4). Consistent with previous observations (Fig. 3B), we detected a discrete sharp band migrating at the expected size of NP that coimmunoprecipitated with L (Fig. 4). L-NP coimmunoprecipitation under these conditions was apparently saturated at 4 μg of transfected pL plasmid (Fig. 4, right-hand gel, lane 5). These results further demonstrate that L and NP are associated, directly or indirectly, in cells in which functional RNPs were reconstituted and can be isolated by different affinity purification strategies.

### NP-bound L forms monodispersed single particles.

**FIG 5:**
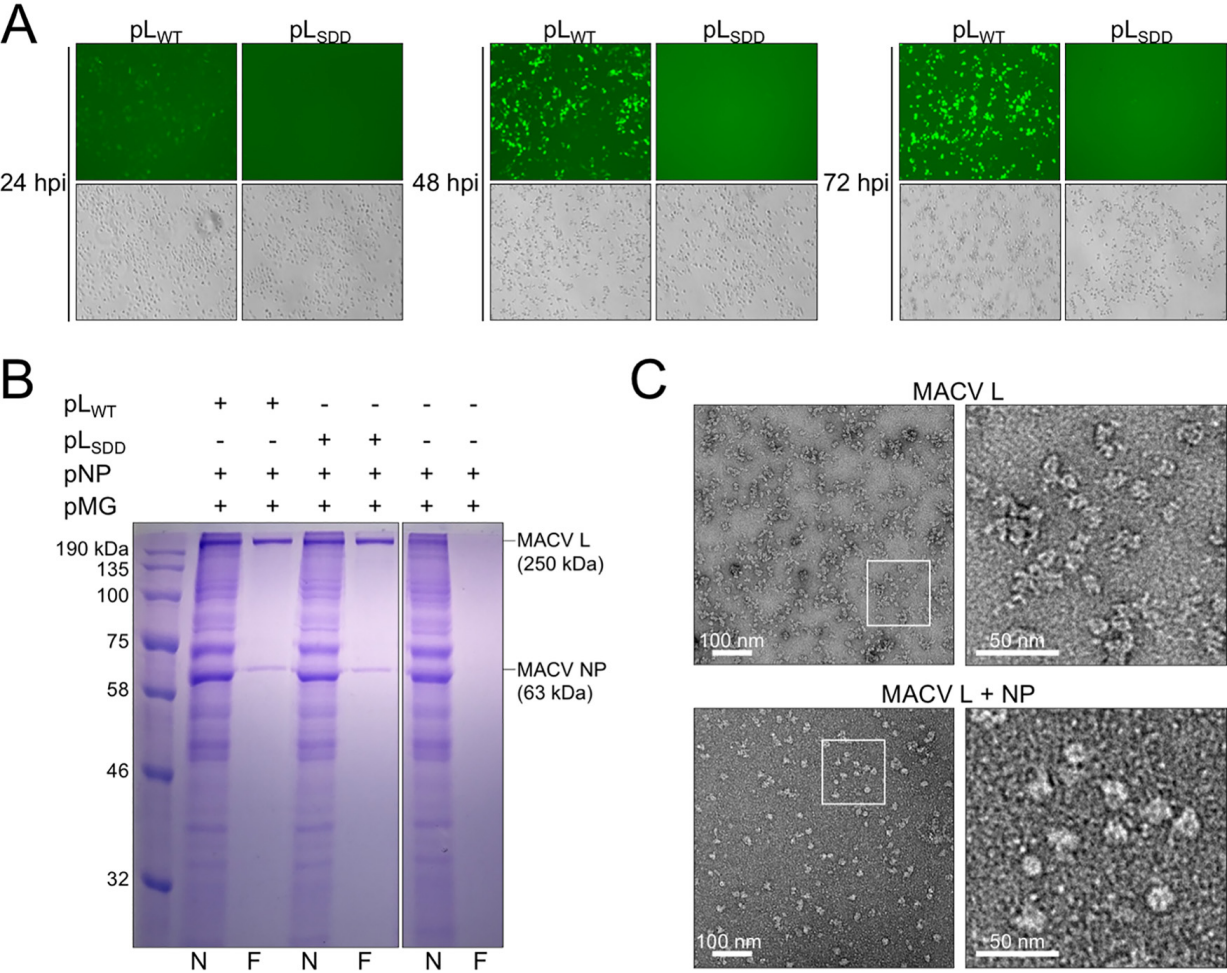
Affinity purification of monomeric L-NP from RNP-expressing cells. (A) Transfected cells expressing functional MACV RNP components accumulate eGFP over the course of 72 h posttransfection (hpt). Cells transfected with wild-type (pL_WT_) or catalytically inactive (pL_SDD_) L, in combination with pNP and pMG, are shown by fluorescence (top) and bright-field (bottom) microscopy images for each condition at 24, 48, and 72 hpt. (B) Affinity purification of MACV L from the cells shown in panel A at 72 hpt. MACV L was purified from BSR-T7 cells as described in Materials and Methods. Lanes: N, final elution from Ni-NTA purification; F, final elution from anti-Flag antibody-mediated purification. (C) Negative-stain EM images of apo-L purified from insect cells and monodispersed L-NP complexes purified from BSR-T7 cells. Selected regions of the micrographs (white outlined boxes) are shown magnified to the right of the L and L-NP particle images.

The copurifying L and NP samples isolated using anti-MACV L polyclonal antibodies (Fig. 4) had fewer copurifying background proteins than samples purified by metal ion affinity chromatography (Fig. 3B). Thus, we sought to utilize the C-terminal Flag epitope tag on MACV L for antibody-mediated affinity purification and competitive elution of the L-NP complex with soluble Flag peptide. BSR-T7 cells were infected with T7 RNAP-expressing vaccinia virus, transfected with the MACV RNP support plasmids, and monitored for eGFP expression for 72 h prior to affinity purification. Cells expressing wild-type MACV L (pL_WT_) gradually accumulated eGFP over 72 h, and this was absent in cells expressing the RdRP catalytic site mutant of L (SDD1328AAA; pL_SDD_) (Fig. 5A). Cell lysates were harvested at 72 h posttransfection (hpt) and subjected to nickel affinity (N) and anti-Flag (F) affinity purifications. Complexes were eluted in high-imidazole buffer or soluble DYKDDDDK(×3) peptide for Ni affinity or anti-Flag purification approaches, respectively.

Elution of antibody-bound protein complexes in the presence of DYKDDDDK(×3) soluble peptide yielded highly pure samples containing both L and NP (Fig. 5B). Wild-type and catalytically inactive L copurified with NP at comparable abundances (Fig. 5B, lanes 3 and 5). Complex elution from cells expressing the minireplicon RNA and NP alone did not yield either L or NP (Fig. 5B, lane 7). In contrast, the imidazole-eluted samples contained background levels of highly abundant copurifying proteins (Fig. 5B, lanes 2, 4, and 6). These results demonstrate that antibody-mediated affinity purification of MACV L from functional RNP-expressing cells yields highly pure L and NP.

Negative-staining and transmission electron microscopy (negative-stain EM) analyses of peptide-eluted samples from wild-type L-expressing cells (Fig. 5B, lane 2) revealed abundant monodispersed particles consistent with other negatively stained images of NSV polymerases (Fig. 5C) (74, 75, 80). Compared with apo-MACV L purified from insect cells, the eluted L-NP sample contained particles that were notably larger and more compacted and globular in shape (Fig. 5C). These observations are generally consistent with heterodimeric L-NP (∼310 kDa) or heterotrimeric L-NP-NP (∼380 kDa) relative to monomeric L alone (∼250 kDa), with possible rearrangements of the flexible N- and C-terminal endonuclease and cap-binding domains of L, respectively (21, 74). These results demonstrate that affinity-purified L and NP form monodispersed particles of slightly larger size than purified L alone.

### MACV RNPs are isolated in a pH-dependent manner.

**FIG 6:**
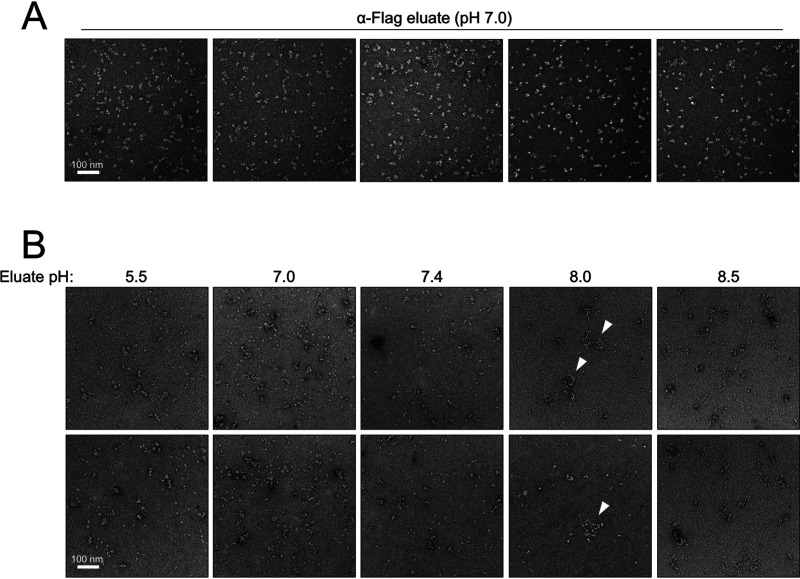
MACV RNPs are purified from BSR-T7 cells in a pH-dependent manner. (A) Representative micrographs of monomeric L-NP particles purified at pH 7.0 by anti-Flag antibody-mediated affinity purification. (B) Representative micrographs of complexes purified from RNP-expressing cells at pH 5.5 to 8.5. RNP-like structures purified at pH 8.0 are indicated by white arrowheads.

During purification optimization, we tested variations of buffer conditions for elution of the L-NP complex. Final elutions performed at neutral pH (7.0) yielded predominantly monodispersed particles, as observed by negative-stain EM (Fig. 5C and 6A). In general, elution of the L-NP complex in solutions with pH ranging from moderately acidic (pH 5.5) to mildly alkaline (pH 8.5) yielded mostly single particles, with some larger-order structures and aggregates observed (Fig. 6B). Samples purified at pH 8.0, however, consistently contained flexible RNP-like structures that were not observed under different buffer conditions (Fig. 6B, fourth column, white arrowheads). These structures had a “beads on a string”-like appearance, resembling shorter variations of purified RNPs from arenavirus (17–19, 81), orthobunyavirus (82, 83), and phlebovirus (84, 85) virions. These observations demonstrate that MACV RNP-like structures can be isolated from BSR-T7 cells in a pH-dependent manner, albeit at lower relative abundances than the monodispersed and larger aggregated complexes.

### MACV RNPs adopt diverse flexible conformations.

**FIG 7:**
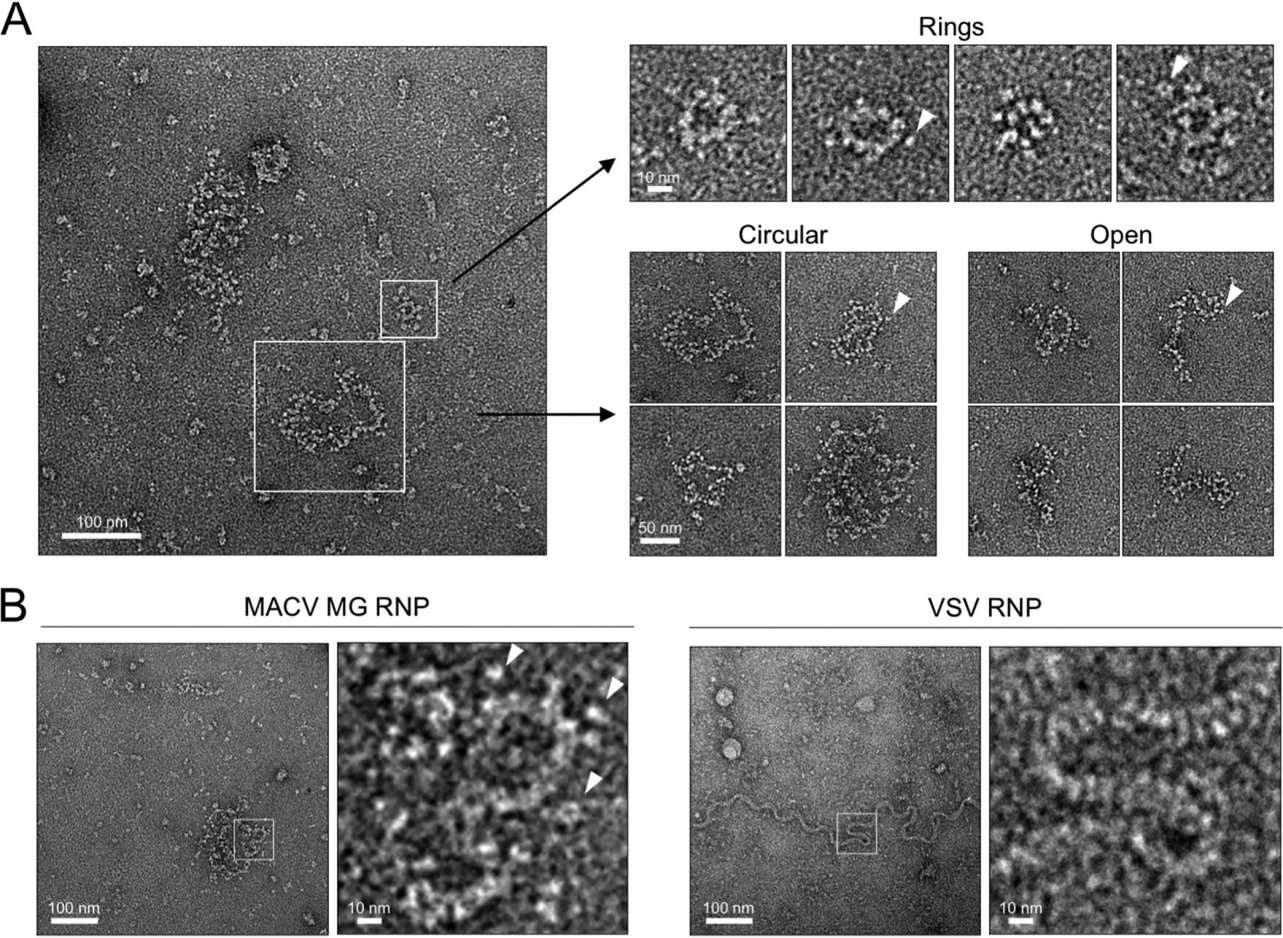
MACV intracellular RNPs adopt diverse flexible structures. (A) The predominant classifications of MACV RNPs isolated from minireplicon-expressing cells. White outlined boxes highlight the different classes of flexible RNPs observed within a representative negative-stain EM micrograph. Examples of the different classes (rings, circular, and open RNPs) are shown to the right of the micrograph. White arrowheads indicate positions of globular particles on the periphery of the RNPs. (B) Comparison of the MACV RNP filaments with the virion RNP of vesicular stomatitis virus (VSV). White outlined boxes indicate regions of flexible RNP curvature, which are magnified and shown to the right of each micrograph. White arrowheads in the magnified boxes indicate positions of globular particles on the periphery of the RNPs.

The MACV RNPs are categorized into three principal architectures: (i) medium to large circularized complexes approximately 100 to 250 nm in diameter, (ii) medium-sized linear complexes approximately 100 to 200 nm long, or (iii) smaller ring-like complexes 30 to 40 nm in diameter decorated with peripheral globular particles (Fig. 7A). The medium and larger complexes were also decorated with globular particles resembling monomeric L (Fig. 5C and 7A). The different RNP classes do not adopt uniform conformations and instead appear highly flexible in solution. This flexibility is highlighted by local curvatures of the RNP filaments, adopting both linear and sharp-angled bends within a single complex (Fig. 7A and B). This inherent filament flexibility is consistent with the RNP structures of other bunyaviruses (17–19, 81, 82, 84–86) and the uncoiled virion RNP of vesicular stomatitis virus (VSV) (Fig. 7B). Each RNP filament is approximately 10 nm in diameter, which is nearly identical to the filament diameters of the Bunyamwera virus (BUNV), Rift Valley fever virus (RVFV), and La Crosse virus (LACV) RNPs (82–85) and earlier EM images of arenavirus RNPs (18, 19, 81). Taken together, these observations strongly suggest that the flexible structures isolated from MACV RNP-expressing cells represent authentic RNPs purified via the affinity epitopes present in L.

### MACV NP forms ring structures.

**FIG 8:**
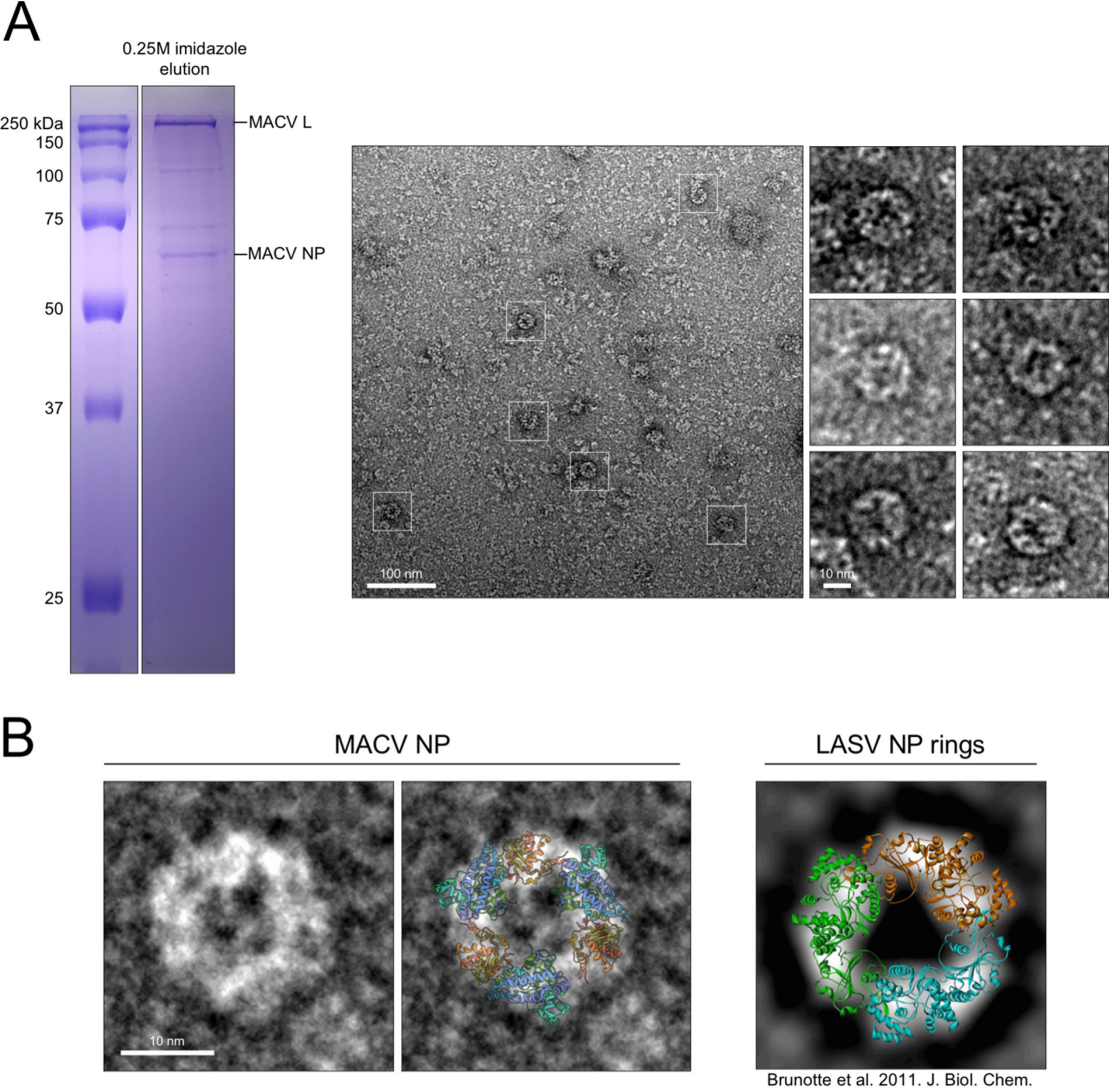
Intracellular NP trimers associate with L. (A) Polyacrylamide-SDS gel from Fig. 3B shown to highlight the metal ion affinity-purified sample visualized in the negative-stain EM micrograph to the right. White boxes highlight representative ring-like structures, with additional magnified structures shown to the right of the micrograph. (B) Comparison of MACV NP rings to the previously characterized RNA-free rings of LASV (right). A cartoon representation of the LASV NP crystal structure (PDB 3MX5) is overlaid on the MACV ring in the right-hand panel. The LASV NP ring image on the far right is modified from Brunotte et al. (CC-BY 4.0) (51).

In addition to the decorated ring-like structures mentioned above, we also documented the formation of smaller circular structures present in electron micrographs of the L-NP complexes purified by Ni affinity chromatography (Fig. 3B and 8A). These smaller rings were approximately 10 to 20 nm in diameter and visually resembled the rings formed by LASV symmetric RNA-free NP trimers in solution (Fig. 8B) (51). Other than aggregates and the crowded molecular background on the EM grids (Fig. 8A), no large or higher-order structures were observed in these samples. The Ni-affinity elution was performed at near-neutral pH (7.4), which could explain the lack of RNP-like complexes observed within these samples. Moreover, the L-NP samples contained additional proteins (Fig. 3B and 8A), suggesting that additional factors could influence the formation of NP rings in solution. Taken together, these findings demonstrate that affinity purification of MACV L from RNP-expressing cells can yield multiple copurifying subcellular complexes, including monodispersed L-NP particles, higher-order RNP structures, and ring-like particles resembling trimeric NP.

## DISCUSSION

**FIG 9:**
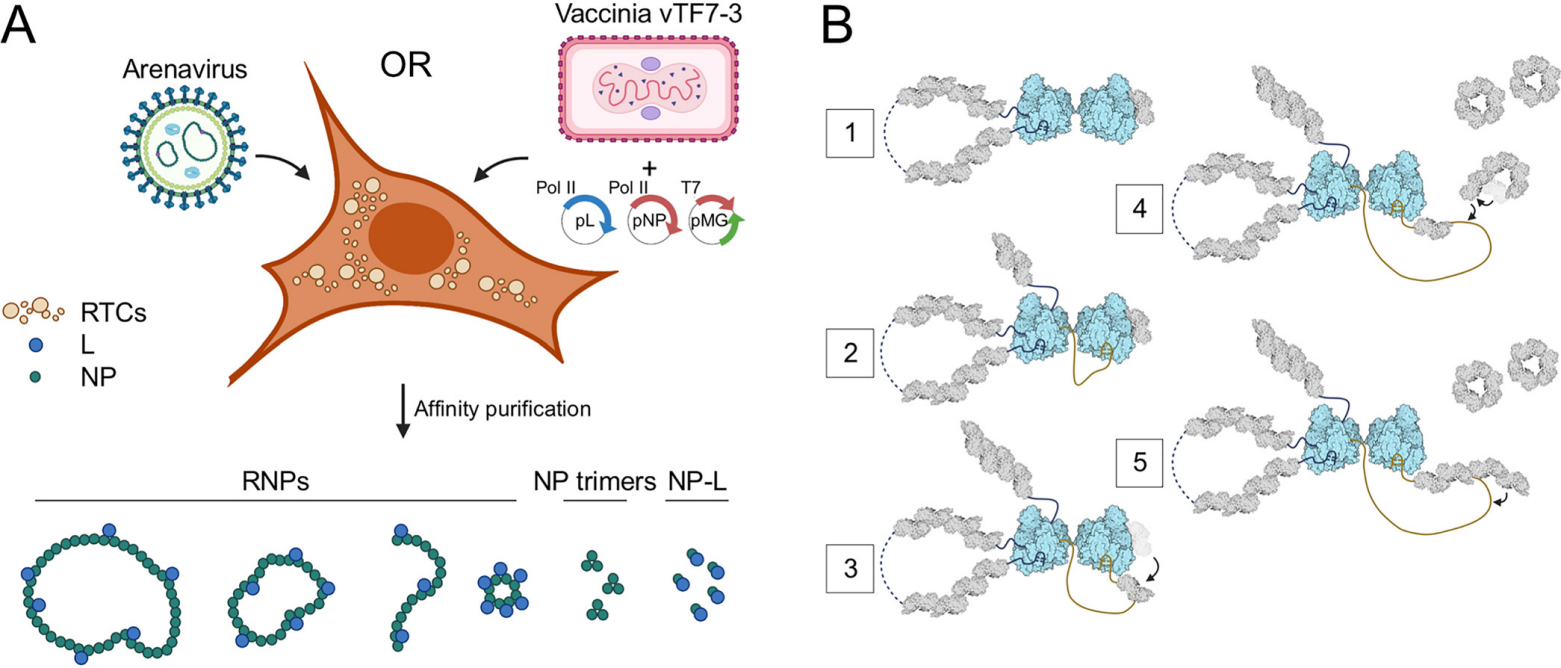
Model of arenavirus subcellular structures formed by L and NP during infection. (A) Illustration highlighting the diverse structures isolated from MACV minireplicon-expressing BSR-T7 cells. (B) Proposed model for the role of a monomeric L-NP complex in RNP replication. (Step 1) A dimeric MACV L structure is formed with one L subunit (left) serving as the active replicase and the other (right) bound to a single NP monomer waiting to receive the nascent RNA from replicase L. (Step 2) As L begins elongation, the nascent 5′ RNA is bound within the 5′ ligand binding pocket of the adjacent inactive L in a “hand-off” coordinated mechanism, stimulating this polymerase for subsequent RNA synthesis on the corresponding 3′ promoter soon to be synthesized (75). (Step 3) NP-bound L facilitates loading of the NP subunit onto the newly synthesized RNA. (Step 4) Soluble NP trimers are triggered to reverse the gating mechanism of RNA binding, allowing for opening of the ring and engagement of the RNA. (Step 5) NP trimers are loaded onto the growing RNP, either three at a time (depicted here) or one by one.

Here, we report a new approach for isolating and characterizing the principal core replication machinery of Machupo virus. We utilized a cell-based minireplicon system for MACV to assess the effects of affinity tags introduced into L and NP on replication and gene expression. Affinity purification of MACV L has yielded unexpected complexes of NP-bound L and RNP-like filamentous structures (Fig. 9A). These complexes, which were purified from mammalian cells expressing an actively replicating MACV L, present a foundation for future biochemical and structural analyses of arenavirus replication mechanisms. Moreover, this system provides an opportunity for further analyses of the monomeric L-NP complex, for which there are no equivalent complexes for any other NSV polymerase or nucleoprotein. This complex may inform our understanding of transient nucleoprotein displacement during RNA synthesis and may provide insight into the coordination of RNP assembly. All prior work on the physicochemical properties of arenavirus RNPs comes from characterization of these complexes from purified virions of mildly pathogenic or nonpathogenic arenaviruses (i.e., Tacaribe virus and Pichinde virus). Detailed analyses of the RNPs from hemorrhagic fever-causing mammarenaviruses are precluded by high biosafety containment requirements—all mammarenaviruses causing severe disease in humans, including MACV, are biosafety level 4 (BSL4) pathogens. Thus, cell-based arenavirus RNP systems are ideal for understanding fundamental mechanisms of replication and may serve as a platform for drug screens and development of targeted antiviral therapeutics.

### L-NP association in cells.

Concrete data and conclusions on the interactions between arenavirus polymerase and nucleoproteins are lacking. It is currently not well understood whether the L-NP interaction is direct, RNA mediated, or both at times. Previous efforts characterizing interactions between L and NP from LCMV and LASV demonstrated that virus-specific RNA was required for copurification of L and NP, suggesting an indirect interaction mediated by RNA binding of both proteins (71). In contrast, expression and coimmunoprecipitation of L and NP in the absence of any viral RNA have demonstrated a direct interaction between the two proteins (72, 73). For Tacaribe virus, the L- and RNA-binding abilities of NP can be uncoupled via point mutagenesis, and direct L binding appears to involve both N- and C-terminal regions of NP (70).

Although we did not specifically test MACV L-NP association in the absence of any expressed viral RNA, the copurification of catalytically inactive L with NP suggests that a processive polymerase with an intact and functional RdRP core is not required for NP engagement. Whether the catalytically inactive mutant is defective in RNA binding is also unknown; however, the identification of secondary promoter binding sites on the solvent-exposed surface of bunyavirus L proteins suggests that a functional active site is not the only mode of RNA recognition by L (21, 87, 88). Thus, the L-NP interaction observed here could also be mediated primarily by RNA, and the L-RNA-NP complexes collapse upon changes in buffer pH *in vitro* to yield monodispersed single particles (Fig. 6). Localized changes in pH could affect the assembly of arenavirus RTC puncta in host cytosol, facilitating the association of newly translated NP with newly synthesized RNA (58, 59, 89). Alternatively, L binding of the 3′ and 5′ untranslated RNA termini—which are required for RNA-mediated L-NP association (71)—could induce a conformation of L that is amenable for NP engagement, such that the L-NP interaction is not directly bridged by an RNA scaffold. This hypothesis is less likely, as virus-specific RNA provided in *trans* failed to induce L-NP interactions (71).

### Functions of the monomeric L-NP complex.

While there is some debate about whether L and NP interact directly during authentic arenavirus infections, the discrete L-NP single-particle complexes identified here suggest that a direct interaction is at least physiologically possible or perhaps mediated by very short RNA sequences. The pH dependence of complex formation may also reflect a biochemical mechanism of RNP assembly, whereby NP monomers preferentially associate with L or NP RNA under neutral or mildly alkaline conditions, respectively. It is likely that arenavirus L and NP must directly associate at some point during the processes of replication and transcription. Other NSVs utilize polymerase cofactors to coordinate transient displacement of nucleoprotein monomers from the template during RNA synthesis (90–93). However, arenaviruses and other NSVs with segmented genomes (e.g., other bunyaviruses and orthomyxoviruses) do not encode analogous polymerase cofactors and thus utilize an inherently different mechanism of nucleoprotein translocation. Whether this is directly mediated by L and which domains of L are involved remain to be investigated. There is also the question of the number of NP subunits per liter—whether L transiently displaces NP one or two subunits at a time, disrupting the interactions between adjacent monomers, or whether the oligomeric NP-NP interactions remain undisturbed as L slides underneath the intact chain to access the liberated RNA template. Future structural efforts aimed at characterizing this monomeric L-NP complex will provide insight into the nature of these interactions.

Alternatively, monomeric L-NP could represent a functionally inactive complex, primed to receive the growing nascent RNA from an adjacent replicase L-RNP. Structural studies of the influenza virus replication machinery suggest that polymerase dimerization facilitates the assembly of progeny RNPs by a product “hand-off” strategy (94). According to this model, the nascent 5′ RNA emerging from an actively replicating polymerase is bound by the adjacent quiescent polymerase, seeding NP deposition onto the newly synthesized RNA and eventually resulting in the formation of a progeny polymerase-bound RNP segment. Perhaps, the adjacent nonreplicating polymerase is preloaded with an NP monomer to initiate this process of RNP assembly. In support of this hypothesis, influenza NP interacts directly with the polymerase PB2 subunit without a requirement for viral RNA, and binding occurs near the site of polymerase dimerization (94–96). Moreover, MACV L dimerization occurs upon promoter RNA binding, and mutation of the dimeric interface region disrupts RNA synthesis *in vitro* (21). Collectively, this suggests that arenaviruses may also participate in a “hand-off” mode of RNP replication and that monomeric L-NP could function on the receiving end of the polymerase replication dimer to begin assembly of the new RNP segment (Fig. 9B). These open questions require future biochemical and structural analyses of purified arenavirus RNP components, like the complexes described in this study.

### Proposed origins and functional consequences of diverse RNP structures.

We observed a heterogeneous population of flexible MACV RNP structures, with filaments of different sizes, shapes, and terminal connectivity (“open” versus “circular”) (Fig. 7). The largest of these complexes could represent NP-bound full-length minireplicon RNA (2,688 nucleotides [nt]), whereas the medium-sized “open” or “circular” segments likely contain shorter RNAs of viral or cellular origin. Minireplicon truncation could occur at secondary structures within the IGR during initial T7 RNAP synthesis. These RNAs would be dead ends for L replication and transcription, as they would not contain the necessary 3′ promoter motif for polymerase binding and initiation (74). However, the presence of correct terminal 5′ sequences could still guide L binding and NP encapsidation of the RNA. The absence of a correct 3′ promoter region would, however, preclude L engagement of both genome termini and pseudocircularization of the RNP (74). This may explain the linear “open” RNPs observed in our samples (Fig. 7A).

The smaller ring-like RNPs (Fig. 7A) could contain truncated 5′ RNAs from the minireplicon that are synthesized during L-mediated replication initiation. Like other bunyaviruses and orthomyxoviruses, 5′ RNA ligands from the viral genome segments are important for stimulating the RNA synthesis activity of arenavirus L (75, 97). Moreover, truncated RNAs corresponding to the 5′ vRNA termini accumulate within influenza virus-infected cells, and these short viral RNAs bind to the polymerase to activate replication in a segment-specific manner (98–100). Whether a related process occurs for arenaviruses or other bunyaviruses remains unknown. Alternatively, these ring-like structures could represent NP-encapsidated host or viral RNAs of a different origin. Unbiased deep sequencing of RNPs purified from minireplicon-expressing and arenavirus-infected host cells will clarify the nature and significance of truncated RNAs and RNP accumulation during infection.

All RNPs appear to be sparsely decorated with globular structures along the periphery of the flexible filaments (Fig. 7, white arrowheads). The identity of these particles is unknown; however, the consistent association with RNP structures implies some degree of biological relevance. It is possible that these particles are monomeric L bound to the surface of RNA-bound NP or directly to exposed RNA sequences within the RNP. Similar structures are present attached to the purified RNPs of other arenaviruses and bunyaviruses (17, 18, 81–85). RNA-bound L in this case would either be stalled along the template, actively synthesizing RNA, or bound to the minireplicon termini.

We also show that expression of the MACV Z protein can inhibit L-mediated replication and transcription of the minireplicon (Fig. 3C). The multifunctional arenavirus Z protein is responsible for coordinating vRNPs into budding virion particles at the cell surface (101) and direct inhibition of L catalytic activity (39). These roles have led to a model whereby Z binding facilitates packaging and egress of transcriptionally primed L with the vRNP segments. The effect of Z binding on the architecture of full RNPs remains unknown. Recent cryo-EM structures of MACV, LASV, and Junin virus L-Z complexes further support the model of Z inhibiting RNA synthesis—either by allosteric inhibition of the L active site (102) or by steric blocking of the RNA product exit channel (103). Our findings presented here offer a system to further probe the regulatory effects of Z using an affinity-tagged module for isolation of Z-bound RNPs (Fig. 3C). Direct visualization of purified Z-bound RNPs by EM could offer insights into the relative position of inhibited L on the RNP and whether Z preferentially inhibits L in a promoter-bound, pre-elongation state or if inhibition is equally likely to occur at internal sites of the minireplicon during active elongation.

The MACV NP ring-like structures (Fig. 8) likely represent the RNA-free “closed” multimeric form of NP, similar to the trimeric rings of other arenavirus NPs (51, 52, 54). These closed rings are hypothesized to function like N^0^-P complexes for nonsegmented NSVs (91). Both structures involve occupation of the nucleoprotein RNA binding site to prevent nonspecific binding of nonviral RNAs in the cell. How these trimeric NP structures form and then rearrange to accept RNA remains a mystery. One possibility is that newly translated NP subunits in proximity, possibly synthesized by polysomes (104), could assemble three at a time in the absence of RNA in the “closed” conformation. Then upon association with L, newly synthesized viral RNA, or a change in local pH, the gating mechanism is triggered to induce opening of the ring and rearrangement of the tertiary structure at the RNA binding site. The three linked NP monomers could be added to the same RNA, like the proposed model for influenza virus RNA encapsidation by preformed NP dimers (105–107), or added sequentially to the same or separate RNAs.

### Model of arenavirus L-NP interactions during replication and transcription.

The above observations have led to a proposed model for the subcellular structures formed by L, NP, and viral RNA during arenavirus infections (Fig. 9). L-mediated replication results in formation of flexible NP-bound vRNP and cRNP structures within the cytoplasmic RTC puncta. Some of these structures are truncated and unsuitable templates for replication or transcription but may play other roles in viral recombination and segmentation, defective particle formation, or stimulation of host immune responses (Fig. 9A). Soluble L is likely bound directly to a subset of NP forming monodispersed single particles—possibly influenced by localized changes in pH—which could function adjacent to an active replicase to seed growth of a newly synthesized RNP. Trimeric NP rings in the cytoplasm adopting a “closed” conformation, incompatible with RNA binding, are prevented from nonspecific encapsidation of host RNAs. An unknown trigger induces the gating conformational switch opening the NP ring and allowing the subunits to bind nascent RNA and assemble into new RNPs (Fig. 9B).

In summary, we present the first structural analysis of subcellular complexes formed by the MACV replication machinery in mammalian cells. These findings provide a foundation for functional and structural analyses of these megadalton assemblies, expanding our understanding of arenavirus L-NP interactions and the assembly of infectious RNPs. Moreover, the cell-based MACV minireplicon presents a unique strategy for advanced biochemical screens to identify essential host factors involved in RNP function and for inhibitors of the RNP assembly process.

## MATERIALS AND METHODS

### Cells and viruses.

BSR-T7/5 cells were used for all MACV RNP experiments and were grown in humidified incubators with 5% CO_2_ at 37 or 34°C as indicated. BSC-40 cells were used for amplification of recombinant vaccinia virus. All mammalian cells were maintained using Dulbecco’s modified Eagle’s medium (DMEM; Corning) supplemented with glucose, l-glutamine, sodium pyruvate, and 10% fetal bovine serum (FBS). Spodoptera frugiperda insect cells (Sf21) were maintained in spinner flasks at 27°C in TC100 medium (Gibco) supplemented with 10% FBS and used for expression of apo-MACV L as described previously (39, 74, 75). T7 RNA polymerase-expressing vaccinia virus (vTF7-3) was amplified on BSC-40 cells and titrated on BSR-T7/5 cells (78, 79). Recombinant vesicular stomatitis virus (VSV) was rescued from cDNA in BSR-T7/5 cells as described previously (108), using T7 RNAP expression plasmids for N, P, L, and G and an eGFP-expressing an infectious genomic cDNA clone of the VSV Indiana serotype. Crude supernatant stocks of VSV from BSR-T7/5 cells infected at a multiplicity of infection (MOI) of 0.1 and harvested at 12 h postinfection were used for the negative-stain EM experiments in Fig. 7B.

### MACV minireplicon cloning and cell expression.

The original pGEM3-based vector for T7 RNAP-driven expression of wild-type MACV L (strain Carvallo; GenBank accession no. AAT40450.1) (74) was modified to include affinity tags at the C terminus of L (Fig. 1B). New coding sequences corresponding to the peptide GG**DYKDDDDK**GGHHHHHH were inserted between the E2209 and stop codon of L, where the Flag and 6×His affinity tags are represented in boldface and underlined text, respectively, separated by glycine linkers. The entire MACV L-Flag-6×His open reading frame was also cloned into an empty pCAGGS vector for the Pol II expression experiments (Fig. 2 and 4). The same cloning steps were performed for a catalytically inactive mutant of L (SDD1328AAA) to generate the pL_SDD_ plasmids. Additionally, wild-type MACV NP (strain Carvallo; GenBank accession no. AAN05426.1) was cloned into empty pGEM3 and pCAGGS vectors (pNP) for the T7 RNAP and Pol II experiments, respectively, and supplemented during transfections with the L-expressing (pL) and minireplicon RNA-expressing (pMG) vectors described below (Fig. 2A). A pcDNA3.1-based vector was used to express NP with a C-terminal V5 affinity tag (GKPIPNPLLGLDST) for the experiments performed in Fig. 3C.

The MACV minireplicon assays were performed essentially as described previously, with minor modifications (39, 74). BSR-T7 cells at 95% confluence in 60-mm-diameter dishes were infected with vaccinia virus vTF7-3 at an MOI of 3 for 1 h at 37°C to supplement endogenous expression levels of T7 RNAP. After infection, plasmids pL, pNP, and pMG were delivered into cells using 20 μl of Lipofectamine 2000 (Invitrogen) in 1.5 ml Opti-MEM following the standard transfection protocol provided by the manufacturer. For the L immunoprecipitation experiments (Fig. 4), the amount of transfected pL plasmid varied from 0 to 8 μg per condition. For the experiments described in Fig. 3B, each 60-mm dish received 2 μg and 4 μg of the pGEM3-based pL and pNP plasmids, respectively, plus 6 μg of the pMG T7 RNAP-driven plasmid for expression of the minireplicon RNA. The same amounts of plasmid were used for the experiments in Fig. 2B, with pGEM3 and pCAGGS plasmids used for the T7 RNAP and Pol II expression conditions, respectively. The plasmid ratios were adjusted for the experiments shown in Fig. 5 based on the immunoprecipitation data, with 4 μg pL, 2 μg pNP, and 6 μg pMG used for each transfection condition. After 4 to 5 h of transfection, the Lipofectamine–Opti-MEM medium was replaced with DMEM supplemented with 2% FBS, and cells were maintained at 34°C. Cells were imaged at 24, 48 (Fig. 2 and 3), and 72 h (Fig. 5) posttransfection by fluorescence microscopy to visually assess eGFP expression from the MACV RNP.

### MACV L antibody production.

Full-length MACV L was purified from insect cells as described previously (74, 75) and used for production of polyclonal sera. Purified MACV L was supplied to the antibody production service company (Covance Research Products) at a final concentration of 0.4 mg/ml in a mixture of 50 mM HEPES, 0.5 M NaCl, and 10% glycerol at pH 7.5. MACV L antisera was produced by a single animal (a New Zealand White rabbit) with an initial subcutaneous injection (250 μg) and three boosts (125 μg each) of purified MACV L over the course of 63 days, followed by production bleeds at 73 and 77 days, respectively.

### L-NP immunoprecipitation and immunoblotting.

Following RNP expression, confluent BSR-T7 cells were removed from each 60-mm dish using sterile cell scrapers, washed twice with 1× phosphate-buffered saline (PBS: 4.3 mM Na_2_HPO_4_, 1.47 mM KH_2_PO_4_, 137 mM NaCl, 2.7 mM KCl [pH 7.4]), and resuspended in 0.75 ml of cold 1× PBS and kept on ice. One-third of the total resuspended cell volume (0.25 ml) was mixed 1:1 (vol/vol) with cold 2× lysis buffer (20 mM Tris-HCl, 132 mM EDTA, 2% Nonidet P-40, 0.8% sodium deoxycholate [pH 7.4]) and incubated on a tube rotator at 4°C for 30 min. After lysis, the ∼0.5-ml crude lysate was centrifuged at maximum speed in a refrigerated tabletop centrifuge for 10 min. The supernatant was collected and used for subsequent MACV L immunoprecipitation and immunoblotting. All buffers were prepared with a supplemented broad-spectrum protease inhibitor cocktail (Sigma).

To immunoprecipitate MACV L from BSR-T7 cells, clarified lysate (489 μl) from the steps described above was incubated with 5 μl of 10% SDS (0.1% final concentration), 5 μl of 100× (10 mg/ml) bovine serum albumin (BSA), and 1 μl of anti-MACV L antiserum (1:500 final dilution). The lysate-antiserum mixture was incubated on a tube rotator at 4°C for 2 h to allow antibody-L binding. The lysate-antibody mixture was then incubated overnight with 25 μl of prewashed protein A-agarose resin (Sigma). After overnight incubation, the protein A resin was pelleted and washed twice with cold 1× PBS supplemented with 0.1% SDS. The final ∼25 μl of washed resin was mixed 1:1 with 1× PBS and used for subsequent immunoblotting analyses.

For immunoblotting analyses, 12.5 μl of each input lysate or resuspended immunoprecipitated sample was mixed with 12.5 μl of 2× SDS loading buffer (100 mM Tris-HCl, 200 mM β-mercaptoethanol, 4% SDS, 0.2% bromophenol blue, 20% glycerol [pH 6.8]) and boiled for 5 min. Sample tubes were centrifuged at maximum speed for 1 min to collect condensation, and 10 μl of each sample was loaded onto a 7.5% polyacrylamide–SDS gel. Gels were run in duplicate for Coomassie staining and immunoblotting using standard equipment (Bio-Rad). For immunoblotting, total separated proteins were transferred from polyacrylamide gels onto hydrated nitrocellulose membranes at 4°C at 300 mA for 2 h. Membranes were incubated by gentle rocking at room temperature for 1 h in a blocking solution of 5% (wt/vol) dried milk in 1× Tris-buffered saline (TBS: 150 mM NaCl, 50 mM Tris [pH 7.4]) with 0.05% (vol/vol) Tween 20 (TBST). Blocking solution was removed, and membranes were incubated overnight in a primary antibody solution of anti-MACV L (1:5,000 dilution in 1× TBST) with 1% (wt/vol) supplemented dried milk (milk-TBST). After overnight incubation, the primary antibody was removed, and blots were washed three times with 1× TBST. A secondary antibody solution of goat anti-rabbit horseradish peroxidase (HRP) conjugate (Invitrogen) diluted 1:10,000 in milk-TBST was added to the blots, and the mixture was incubated at room temperature for 1 h. The secondary solution was removed, and the blots were washed twice with 1× TBST and once with 1× TBS to remove trace amounts of detergent. Blots were developed with standard HRP chemiluminescence substrate reagents (Pierce) and imaged using an Amersham Imager 600 (GE Life Sciences).

### L affinity purification.

Cell lysates were prepared from vaccinia virus-infected and -transfected BSR-T7 cells following the same initial steps outlined above for the MACV L immunoprecipitation. Following centrifugal clarification, 0.75 ml of lysate was combined with 25 μl of prewashed anti-DYKDDDDK antibody-conjugated affinity resin (Pierce) or 25 μl of prewashed Ni-nitrilotriacetic acid (NTA) agarose (Qiagen). Lysate-resin mixtures were incubated on a tube rotator at 4°C overnight. Resins were pelleted by centrifugation in a refrigerated tabletop centrifuge at ∼200 × *g* for 1 min, and the unbound lysate fraction was removed. Resins were washed three times with 1× PBS supplemented with 0.1% SDS. The wash buffer for Ni-NTA purifications included 20 mM imidazole to reduce weak or nonspecific binding events. After the third wash, 150 μl of elution buffer [1× TBS supplemented with 10% glycerol and either 0.25 M imidazole or 250 μg/ml of DYKDDDDK(×3) synthetic peptide for Ni-NTA and anti-DYKDDDDK elutions, respectively] was added to each corresponding pelleted resin. The final elution pH was typically near neutral at 7.0 to 7.4; however, a broader range was tested for the experiments presented in Fig. 6 and 7. Samples were incubated on a tube rotator at room temperature for 30 min to elute resin-bound L. All buffers were prepared with a supplemented broad-spectrum protease inhibitor cocktail (Sigma).

### Negative staining and electron microscopy.

Negative staining of the L-NP complexes was performed as described previously (75). Purified samples at approximately 0.02 to 0.05 mg/ml were applied to glow-discharged carbon-coated copper grids (Ted Pella) and stained with 0.75% (wt/vol) uranyl formate. Samples were imaged using a Philips CM10 microscope operated at 100 kV with a tungsten filament and UltraScan 894 charge-coupled device (CCD) camera (Gatan), or a Tecnai T12 microscope operated at 120 kV with a lanthanum hexaboride filament and UltraScan 895 CCD camera (Gatan). Images were collected at a magnification of 54,000× with an approximate defocus of −1.5 μm.
